# HPV knowledge and acceptance of HPV vaccination among men who have sex with men (MSM) in Germany: a multicenter, observational, cross-sectional study

**DOI:** 10.1186/s12889-025-25746-x

**Published:** 2025-12-12

**Authors:** Cornelia Wähner, Lisa M. Lang, Maher Almahfoud, Daniel Beer, Stefan Esser, Matthias C. Müller, Nils Postel, Anja Potthoff, Stephan Schneeweiß, Sven Schellberg, Agnes Luzak

**Affiliations:** 1 Department of Medical Affairs, MSD Sharp & Dohme GmbH, Levelingstraße 4a, München, 81673 Germany; 2 Department of Market Access, MSD Sharp & Dohme GmbH, Munich, Germany; 3 1st Department of Medicine, Division of Infectious Diseases, University Medical Center Hamburg-Eppendorf, Hamburg, Germany; 4 Praxis/Labor Dr. med. Heribert Knechten, PZB Aachen, Aachen, Germany; 5 Clinic and Polyclinic for Dermatology and Venereology and Allergology, University Hospital Essen, Essen, Germany; 6 Department of Infection Medicine, Medical Service Center Clotten, Freiburg im Breisgau, Germany; 7 prinzmed, Practice for Infectiology, Munich, Germany; 8 Interdisciplinary Immunological Outpatient Clinic, Sexual Health and Medicine, Department of Dermatology, Venereology and Allergology, St. Elisabeth Hospital Bochum, WIR – Walk in Ruhr, Bochum, Germany; 9 Praxis Hohenstaufenring, Cologne, Germany; 10 Specialist in general medicine and sexual health, Novopraxis GbR, Berlin, Germany

**Keywords:** MSM, HPV knowledge, HPV vaccination acceptance, HPV vaccination uptake

## Abstract

**Background:**

Men who have sex with men (MSM), especially those living with HIV, have a high prevalence of HPV infections that might lead to HPV-related diseases, including penile, oropharyngeal and anal carcinomas. Currently, beyond recommendations to get vaccinated during childhood, adult MSM are not included in the German national HPV vaccination recommendation. The aim of this cross-sectional study was to assess knowledge of HPV and HPV vaccination, and further acceptance and uptake of HPV vaccination among MSM in Germany.

**Methods:**

Participants were recruited between 10/2023 and 07/2024 at 10 German study sites specialized on sexual and/or men’s health. Main inclusion criteria were being MSM, being aged 18–45 years and a German health insurance. Data were obtained through an electronic questionnaire and analyzed descriptively. 20 HPV and 5 HPV vaccination knowledge questions were evaluated using sum scores, with each correct answer scoring 1 point.

**Results:**

929 participants, aged 18–45 years (median 31), were included in the analysis. 122 (13.1%) were living with HIV and 717 (77.2%) used HIV-PrEP. 731 (78.7%) had prior knowledge of HPV, and 604 (65.0%) were aware of HPV vaccination. Mean sum scores for HPV knowledge (*N* = 731) and HPV vaccination knowledge questions (*N* = 604) were 13.3 ± 4.3 and 3.0 ± 1.3, respectively. Among those aware of HPV vaccination, 377 (62.4%) accepted vaccination (defined as already vaccinated (*N* = 204) or intending to get vaccinated (*N* = 173), and 223 (36.9%) participants were hesitant. Main reasons for hesitancy included a lack of information and no mandatory reimbursement of adult HPV vaccination costs. For MSM who intended to get vaccinated, payment out of pocket and missing physician recommendations were the main reasons for not being vaccinated yet. Among all analyzed MSM (*N* = 929), 127 (13.7%) completed a full vaccination schedule (21% within those aware of HPV vaccination).

**Conclusion:**

Despite almost 80% participants being aware of HPV, vaccination uptake was low among all participants. More information about HPV, i.e., through physicians and social networks, might support informed decision-making. Further, facilitating access by offering reimbursement options for vaccination could potentially be a factor to help increase HPV vaccination uptake among MSM in Germany.

**Supplementary Information:**

The online version contains supplementary material available at 10.1186/s12889-025-25746-x.

## Introduction

 Human papillomaviruses (HPV) are the most common sexually transmitted pathogens, with nearly 100% of sexually active individuals becoming infected at least once during lifetime [1]. Persistent HPV infection can lead to various diseases, including genital warts and several types of cancer in both men and women [2]. In men, approximately 85% of anal carcinomas, 40% of oropharyngeal carcinomas, and about 45% of penile carcinomas are attributed to persistent HPV infection [3]. Especially men who have sex with men (MSM), particularly those living with HIV, have a high prevalence of HPV infection and related diseases [4, 5]. HPV vaccination could prevent certain HPV-associated diseases in this population.

In Germany, HPV vaccination has been recommended by the German Standing Committee on Vaccination (STIKO, Ständige Impfkommission) for girls only since 2007. Since 2018, HPV vaccination has been recommended in a gender-neutral manner for all adolescents aged 9–14, with a catch-up option up to age 17 [6]. Despite this universal recommendation, vaccination uptake among boys remains low (in 2023, only 26.4% were vaccinated by age 18) and vaccination of adults is currently not recommended by STIKO [6, 7]. There is also no specific HPV vaccination recommendation for MSM, unlike in other countries such as Australia, Canada, Spain, UK, Italy and France [8–13]. However, a nationally binding recommendation by the STIKO is a prerequisite for mandatory reimbursement of the vaccination by the statutory health insurers. Nevertheless, some health insurance providers voluntarily refund vaccination costs up to age 26, which MSM can also benefit from [14]. In Germany, approximately 2.1% of men aged 18–75 identify as MSM, and 1.4% identify as bisexual, with higher proportions observed among younger men (5.0% MSM and 2.0% bisexual at ages 21–25) [15].

At present, evidence on HPV knowledge, vaccination willingness and uptake among MSM in Germany is limited. To address this gap, we conducted this multicenter, observational, cross-sectional study among MSM aged 18–45 years in Germany. The primary objectives were to (1) assess knowledge of HPV and HPV vaccination; and (2) evaluate acceptance of HPV vaccination. Secondary objectives were to (3) determine HPV vaccination uptake and (4) describe the main outcomes (sum scores of HPV and HPV vaccination knowledge), acceptance, and uptake by age groups (18–26 years, 27–45 years) and HIV status.

## Methods

### Study design

This multicenter, observational, cross-sectional study is focusing on MSM aged 18 to 45 years, aiming to assess knowledge and acceptance of HPV vaccination, as well as HPV vaccination uptake. A convenience sampling approach was used. Data were collected at 10 public physicians’ offices specialized in men’s health, treatment and prevention of sexually transmitted diseases (STDs) including HIV, and pre-exposure prophylaxis (HIV-PrEP) across Germany. Participant recruitment and study participation occurred by participants completing one electronic questionnaire during a single routine physician visit after providing informed consent for study participation. Participants were provided with a 15€ gift card as compensation for their time invested.

### Participant recruitment

The study was designed to enroll a maximum of 1,000 participants. During routine physician visits, potential participants were informed about the study, and their eligibility was assessed through a screening interview conducted by their treating physician. The interview included the assessment of inclusion criteria:

1. having signed an informed consent form (ICF) that included data protection consent,
2. being MSM (i.e., defined as having sex exclusively with men, or with men and women),
3. being aged between 18 and 45 years,
4. being a permanent resident in Germany for at least three years,
5. being insured by a German health insurance company,

and additionally, information on their HIV status.

Participants were divided into two age groups: 18–26 years and 27–45 years. The initial recruitment employed balanced recruitment with a target quota, aiming for an equal 50:50 distribution between age groups (18–26 and 27–45). However, due to difficulties in enrolling young MSM, an adaptive recruitment strategy was applied.

Participants were excluded if they were unable to read and understand a questionnaire in German or English. Recruitment took place between October 2023 and end of July 2024.

### Data collection

Data was collected through an electronic questionnaire, available in German and English, administered on a tablet at the study sites, which participants completed directly after being included in the study. The questionnaire was developed in German and professionally translated into English. Accuracy and conceptual equivalence were ensured through additional review and refinement of the English version, without formal back-translation. The questionnaire took approximately 15 min to complete and included questions on socio-demographics, sexual behavior, HPV and HPV vaccination knowledge, acceptance and uptake of HPV vaccination, as well as the impact of reimbursement on vaccination acceptance.

The questionnaire required participants to answer each question before moving on to the next. 983 participants were screened via screening interview of whom 12 were excluded due to violation of inclusion or exclusion criteria,14 prematurely withdrew from the study without data usage and 28 discontinued before answering the question regarding HPV knowledge. Being contradictory to the required inclusion criterion that participants are having sex with men, participants were not included in the analysis population if they answered to be sexually active in the past 12 months but their sexual preference in the last 12 months were never men. Ultimately, 929 MSM were included in the analysis population.

**Fig. 1:**
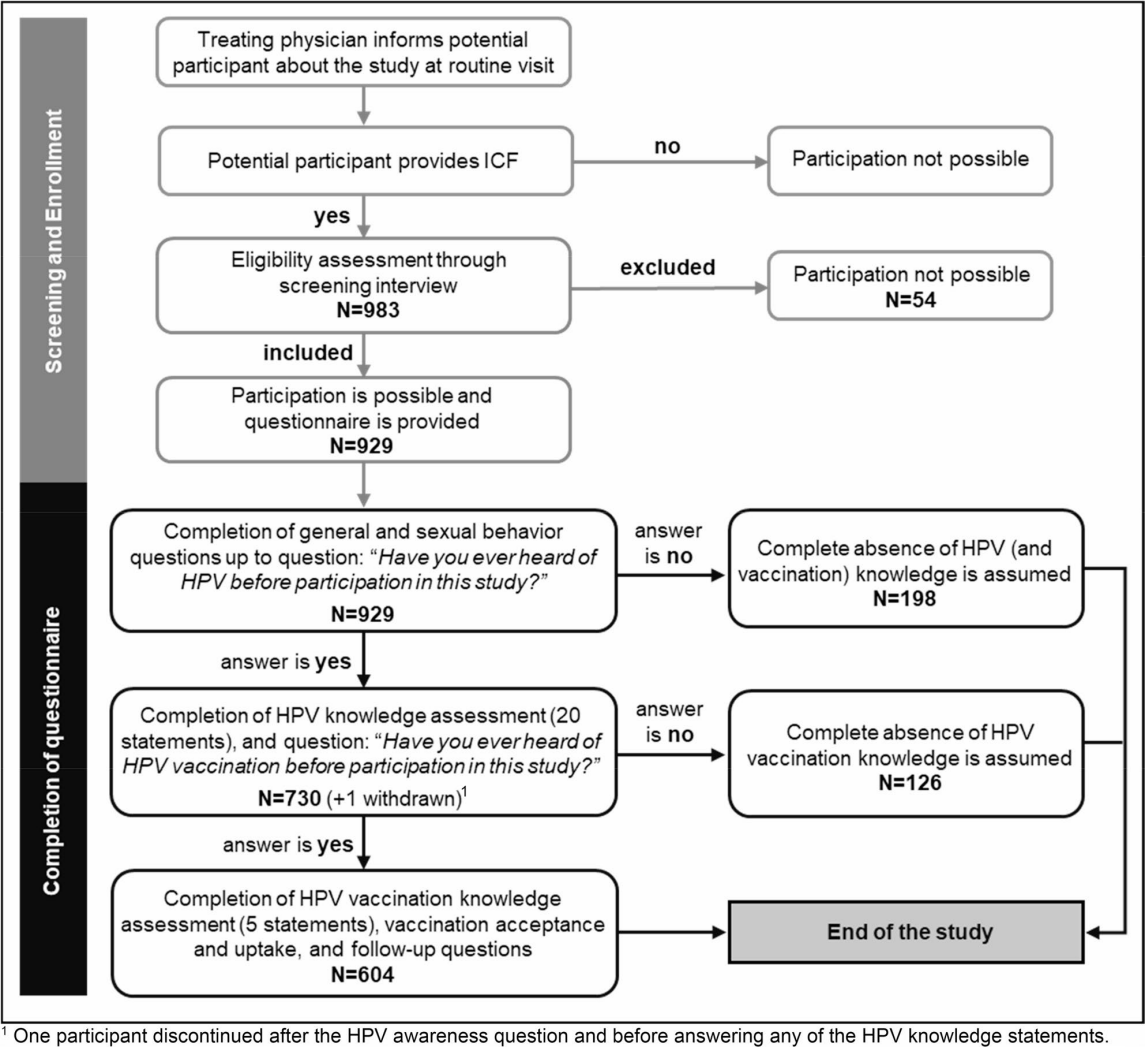
Study flow chart

An overview is displayed in Fig. 1, and the full questionnaire can be found in the supplement.

### Study measures

The questionnaire incorporated branching logic based on participants’ responses to key questions (shown in Fig. 1). Participants who indicated no prior awareness of HPV before the study were not presented with the section assessing HPV knowledge, meaning the questionnaire ended. Similarly, only participants who reported being aware of the HPV vaccination received questions related to HPV vaccination knowledge, acceptance and uptake. Clear descriptions of the denominators were applied to specify the exact subgroups the main outcomes refer to.

#### HPV and HPV vaccination knowledge

Knowledge about HPV was assessed with 20 statements (e.g. “HPV is very rare“, “Men cannot get HPV“, “HPV can cause cancer in men“, etc.) and knowledge about HPV vaccination with 5 statements (e.g. “You can cure HPV by getting the HPV vaccine“, “HPV vaccines protect against all STDs“, etc.) that could be answered by “true/false/I don´t know” (see supplement, Questionnaire: Questions 20 and 29). To avoid missing data within the scale questions, the participants needed to choose one of the three answer options for each single question in order to proceed with the next scale question. The scale items were translated from a published questionnaire used in a study by Kesten et al. which investigated young MSM’s knowledge and attitudes towards HPV [16]. In that study, the authors drew on validated scales developed by Perez et al. [17] and modified them for MSM, with input from an expert panel including a key stakeholder group, and MSM focus groups.

#### HPV vaccination acceptance

HPV vaccination acceptance was defined in the following three categories, which were based on the participants’ answers to their attitudes regarding HPV vaccination (based on questions 30 (“Have you already been vaccinated against HPV with at least one dose?”) and 37 (“If you have not yet been vaccinated against HPV, how do you feel about HPV vaccination?”) of the questionnaire, see supplement, questionnaire):

1. Accepts HPV vaccination, including.

- MSM who are already vaccinated with at least one dose.
- MSM who intend to get vaccinated.

2) Is hesitant, including.

- MSM who are undecided regarding HPV vaccination.
- MSM who never thought about getting the vaccination against HPV.

3) Does not accept HPV vaccination, including.

- MSM who refuse HPV vaccination.

In this study, “accepts HPV vaccination”, was defined by summarizing two measures (vaccination status and intention) to reflect a general openness toward HPV vaccination. “Is hesitant” denotes non-acceptance without outright refusal, including participants who are undecided and those who have never considered getting vaccinated against HPV.

#### HPV vaccination uptake

HPV vaccination uptake was defined as MSM who had received a full HPV vaccination schedule, depending on the number of doses received at the respective age. A full vaccination schedule was defined based on the European Union Summary of Product Characteristics (EU SmPC) [18]. Participants initiating the series at age ≤ 14 years were considered complete after 2 doses; those initiating at ≥ 15 years required 3 doses. Vaccination uptake was assessed (A) for the total analysis population (including those unaware of HPV vaccination, assuming participants unaware are unvaccinated), leading to a lower-bound estimate and (B) as a sensitivity analysis, for those aware of HPV vaccination.

#### Subgroup analyses of main outcomes

The main outcomes HPV and HPV vaccination knowledge, acceptance of HPV vaccination (stratified by does accept, does not accept, is hesitant) and HPV vaccination uptake were further stratified by age group (18–26 years vs. 27–45 years) and HIV status (HIV positive, HIV negative).

Furthermore, the reasons for non-vaccination within those aware of HPV vaccination were evaluated, as well as the main sources of HPV and HPV vaccination information.

### Statistical analysis

All participants were pooled into one analysis population. All data collected in the screening interview and the questionnaire was evaluated using standard descriptive statistics with the statistical software SAS 9.4: continuous variables were described by arithmetic mean, standard deviation (SD), minimum, 1 st quartile, median, 3rd quartile, and maximum. Categorical variables are described by absolute numbers (N) and percentages (%), including a category for missing values. The objectives were analyzed descriptively only, and therefore, no specific statistical hypotheses were defined. Since this was a purely descriptive study without hypothesis testing, a formal sample size or power calculation was not required; however, a sufficient sample size of approximately 1,000 participants was planned to ensure adequate precision and representativeness of the descriptive results. Considering secondary endpoints, with 1000 subjects, differences in subgroups in the range of an effect size between 18% and 30% for sizes of groups > 100 subjects (type-I error rate 5%, power 80%) could be detected.

To analyze [1] HPV and HPV vaccination knowledge, mean sum-scores with the respective 95% confidence intervals (CI) calculated using Student’s t-statistics were presented.

To derive the sum-score the primary scoring rule was: each correct answer received a score of 1, and each false answer or “I do not know” answer received a score of 0. The number of correct answers was summed up, leading to a score ranging from 0 to 20 points for “knowledge of HPV” and from 0 to 5 points for “knowledge of HPV vaccination”. Higher scores represent higher knowledge, i.e., more correct answers. A sensitivity analysis was conducted on the summary scores for HPV and HPV vaccination knowledge to assess the impact of the scoring method. In the sensitivity scoring, incorrect answers were scored as −1, which penalizes guessing and favoring participants who chose “I do not know” instead of risking a wrong answer.

For [2] HPV vaccination acceptance and [3] HPV vaccination uptake, the respective proportions were given together with 95% CIs calculated using a binomial distribution, i.e., exact Clopper-Pearson method. HPV vaccination uptake was calculated based on (A) the total analysis population (including those unaware of HPV vaccination, assuming participants unaware are unvaccinated) and (B) for those aware of HPV vaccination.

For [4], subgroup analyses were performed for age groups (18–26 years, 27–45 years) and HIV status (HIV+, HIV-). The group of participants that reported not being tested for HIV were not considered in HIV subgroup analyses (*N* = 11).

## Results

### Study population

A total of 983 people were considered for recruitment into the study. Of those, 12 (1.2%) did not meet the inclusion criteria, 14 (1.4%) withdrew their consent for data usage, and 28 (2.8%) ended the questionnaire prematurely. The final analysis population consisted of 929 MSM.

### Participant characteristics

Sociodemographic characteristics are shown in Table 1. Participants were on average 31.2 ± 6.6 years old. Most participants (98.8%) had previously been tested for HIV, with the majority tested negative (85.7%). Only 11 participants (1.2%) had never been tested. Over half of the participants resided in cities with more than 500,000 inhabitants (51.2%). Regarding education, 44.0% had completed a university degree and 26.6% had completed vocational training. More than two-thirds were employed full-time and over 90% reported being covered by a German statutory health insurance.

**Table 1:** Baseline characteristics

Baseline Characteristics	*N* (%)
*Analysis Population*	*929 (100)*
Mean age in years (SD)	31.2 (6.6)
**Current place of residence**	
Residence with more than 500,000 inhabitants	476 (51.2)
Residence with 100,000–500,000 inhabitants	232 (25.0)
Residence with 20,000–100,000 inhabitants	111 (12.0)
Residence with less than 20,000 inhabitants	110 (11.8)
**Highest educational attainment**	
No school-leaving qualification	2 (0.2)
Still in school/university/vocational training	55 (5.9)
Secondary school diploma (9 years of school)	27 (2.9)
Secondary school diploma (10 years of school)	74 (8.0)
General qualification for university entrance	115 (12.4)
Completion of vocational-in-company training (apprenticeship)/vocational-school training (vocational college, college)	247 (26.6)
University/college degree	409 (44.0)
**Employment status**	
Full-time employment	632 (68.0)
Part-time employment	106 (11.4)
Marginally employed, 520-euro job, mini-job	41 (4.4)
In a vocational training	34 (3.7)
Not employed (including: pupils or students, jobseeker, early retirees)	116 (12.5)
**German health insurance**	
Statutory, compulsorily insured	741 (79.8)
Statutory, voluntarily insured	127 (13.7)
Private	61 (6.6)

### Sexual behavior

Data on sexual behavior is presented in the supplement, Table A1. Almost all participants (98.3%) reported being sexually active in the past 12 months. Among them, 43.5% reported having had between 2 and 10 sexual partners, 23.4% more than 10 partners, and 25.0% more than 20 partners during this period. The most common sexual practice was oral sex (98.5%). Condom usage varied, with 17.6% reporting "Never", 43.8% "Rarely", and 32.4% "Mostly". Approximately three-quarters (77.2%) of the MSM reported having had a HIV-PrEP prescription in the past. Among 730 participants that have at least heard of HPV before, their self-estimated risk of acquiring HPV was rated high by 24.1%, moderate by 34.4%, and low by 22.3% of participants (see supplement, Table A4).

### HPV and HPV vaccination knowledge

A total of 198 participants (21.3%) reported that they had never heard of HPV prior to the study and therefore did not receive further questions. One participant discontinued the questionnaire after completing this question, therefore only the remaining 730 participants (78.7%) who were aware of HPV completed the HPV knowledge assessment. 

The mean summary score was 13.3 ± 4.3 (range: 0-20; Figure 2A); with a median of 14 correctly answered questions. 25% of participants answered at least 17 questions correctly (this was true for the total population and within each subgroup shown). Among those aware of HPV, 126 participants (13.6%) had never heard of the HPV vaccination, leading the questionnaire to end with this question. The remaining 604 participants were evaluable for the HPV vaccination knowledge assessment, achieving a mean summary score of 3.0 ± 1.3 (range: 0-5; Figure 2B); with a median of 3 correctly answered questions and 25% of participants answering at least 4 out of 5 questions correctly (this was true for the total population and within each subgroup shown, please see supplement, Table A2).

**Fig. 2:**
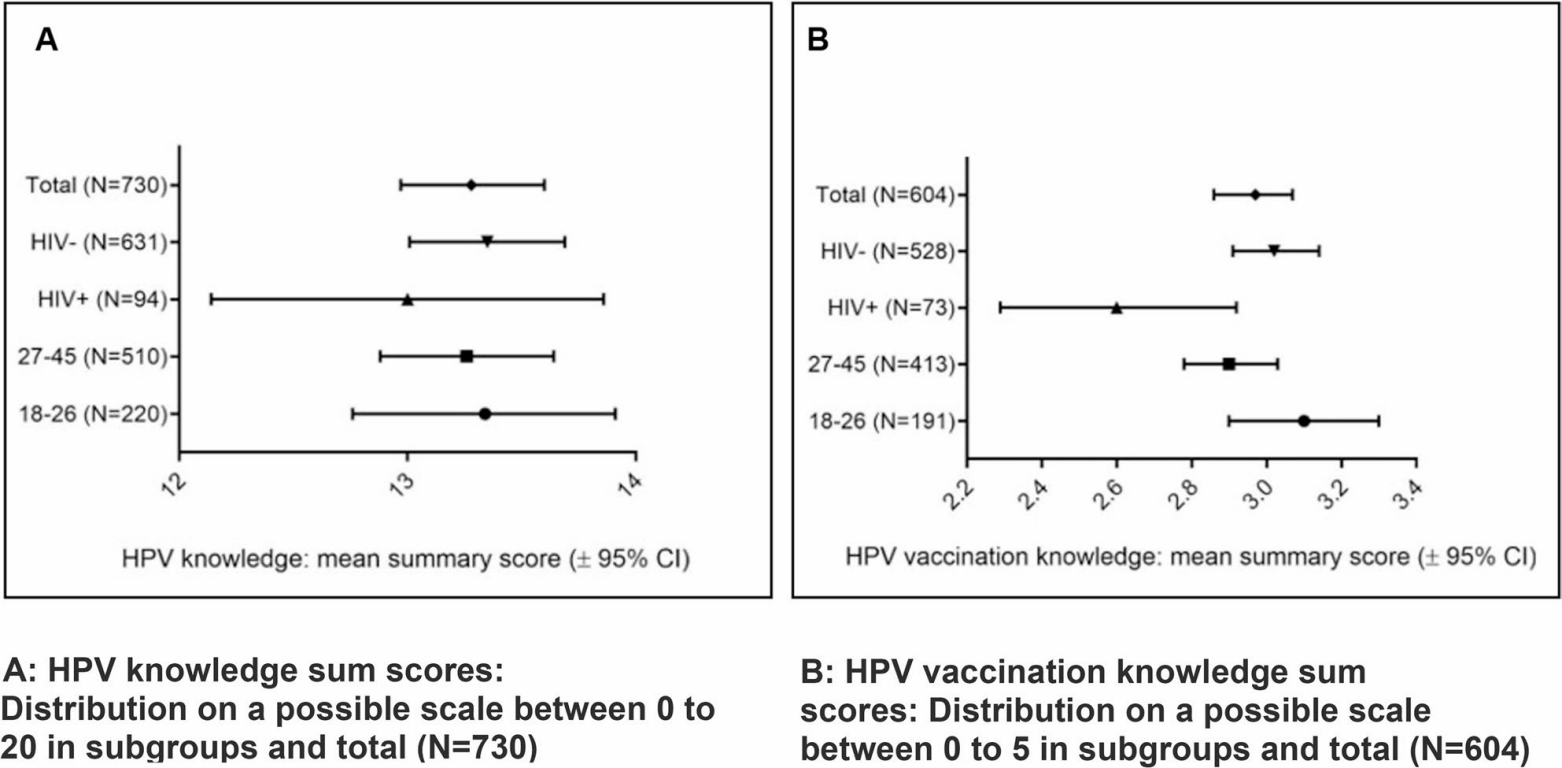
Knowledge sum scores

In a sensitivity analysis in which wrong answers were scored as -1 and thus reducing scores for participants who might have just guessed the answers, mean summary scores (±SD) were 10.9 ±5.2 for HPV knowledge (N=730) and 2.4 ±1.8 for HPV vaccination knowledge (N=604) (see supplement, Table A3). 

### HPV vaccination acceptance and uptake

HPV vaccination acceptance was assessed among study participants who were aware of HPV vaccination prior to the study (*N* = 604). The majority with 62.4% (*N* = 377) accepted HPV vaccination, meaning 204 participants received at least one dose and 173 intended to get vaccinated. While acceptance among HIV-positive and HIV-negative participants was similar, about 80% of the younger cohort (ages 18–26) accepted the vaccination, whereas only around 55% of the 27–45-year-olds did so. In addition, 36.9% (*N* = 223) of participants were hesitant about HPV vaccination, and 0.7% (*N* = 4) refused it (see Table 2).

**Table 2:** HPV vaccination acceptance

HPV vaccination acceptance	Total population *N* (%) [95% CI]	Age in years, *N* (%) [95% CI]		HIV status, *N* (%) [95% CI]	
		18–26	27–45	HIV+	HIV-
*Among those aware of HPV vaccination*	*604 (100)*	*191 (100)*	*413 (100)*	*73 (100)*	*528 (100)*
Accepts HPV vaccination ^1^	**377 (62.4)** [58.4; 66.3]	**151 (79.1)** [72.6; 84.6]	**226 (54.7)** [49.8; 59.6]	**46 (63.0)** [50.9; 74.0]	**328 (62.1)** [57.8; 66.3]
Is hesitant regarding HPV vaccination ^2^	**223 (36.9)** [33.1; 41.1]	**37 (19.4)** [14.0; 25.7]	**186 (45.0)** [40.2; 50.0]	**27 (37.0)** [26.0; 49.1]	**196 (37.1)** [33.0; 41.4]
Does not accept HPV vaccination	**4 (0.7)** [0.2; 1.7]	**3 (1.6)** [0.3; 4.5]	**1 (0.2)** [0.0; 1.3]	**0 (0)** -	**4 (0.8)** [0.2; 1.9]

^1^ Acceptance of HPV vaccination is a composite of those being vaccinated with at least 1 dose (*N* = 204) and those who intend to get vaccinated (*N* = 173)

^2^ Hesitancy regarding HPV vaccination is a composite of those being undecided (*N* = 102) and those who never thought about it (*N* = 121)

Among all participants in the analysis population (*N* = 929), 13.7% (*N* = 127) reported being fully vaccinated (assuming those unaware of HPV vaccination are unvaccinated). When considering only those who had heard about HPV vaccination (*N* = 604), 21.0% (*N* = 127) reported being fully vaccinated. A higher proportion of younger participants completed the full vaccination series than the older group (29.3% vs. 17.2% within those aware of HPV; 20.0% vs. 10.9% within the total population). HIV status was similar in both groups (see Table 3).

**Table 3:** Uptake of HPV vaccination

HPV vaccination uptake	Total population *N* (%) [95% CI]	Age in years, *N* (%) [95% CI]		HIV status, *N* (%) [95% CI]	
		18–26	27–45	HIV+	HIV-
*Calculated considering those aware of HPV vaccination*	*604 (100)*	*191 (100)*	*413 (100)*	*73 (100)*	*528 (100)*
Participants that received a full HPV vaccination schedule	**127 (21.0)** [17.8; 24.5]	**56 (29.3)** [23.0; 36.3]	**71 (17.2)** [13.7; 21.2]	**12 (16.4)** [8.8; 27.0]	**115 (21.8)** [18.3; 25.6]
*Calculated considering the total population* ^1^	*929 (100)*	*280 (100)*	*649 (100)*	*122 (100)*	*796 (100)*
Participants that received a full HPV vaccination schedule	**127 (13.7)** [11.5; 16.0]	**56 (20.0)** [15.5; 25.2]	**71 (10.9)** [8.6; 13.6]	**12 (9.8)** [5.2; 16.6]	**115 (14.5)** [12.1; 17.1]

^1^ Assuming participants unaware of HPV vaccination are unvaccinated

### Reasons for non-vaccination

Dependent on their HPV vaccination acceptance, participants received follow-up questions on the reasons (Table 4.). Among participants who intended to get vaccinated (*N* = 173), 42.8% reported not being vaccinated yet due to out-of-pocket payment. 20.8% were missing a recommendation by their physician. Among participants who were hesitant about HPV vaccination (*N* = 102), the primary reasons for their hesitancy (multiple responses allowed) included insufficient information about the vaccination (42.2%), high cost (40.2%), and the absence of a national recommendation for HPV vaccination in MSM (24.5%).

**Table 4:** Reasons for non-vaccination: Follow-Up questions on HPV vaccination acceptance [Only for participants who had heard about HPV vaccination (*N*=604) and dependent on whether they were vaccinated with at least one dose (*N*=204), they intend to get vaccinated (*N*=173), they were undecided (*N*=102) , they never thought about the vaccination (*N*=121) or they refuse vaccination (*N*=4)]

Reasons for non-vaccination	N (%)
*Within those vaccinated with at least 1 dose*	*204 (100)*
**Age when vaccinated**	
9-14 years	6 (2.9)
15-17 years	11 (5.4)
18-26 years	125 (61.3)
27-45 years	62 (30.4)
**Who paid for the vaccination**	
I was vaccinated before my 18th birthday and the vaccination was charged to my health insurance card	22 (10.8)
I was over 18 years old and paid for the vaccination privately but was subsequently reimbursed by my health insurance	126 (61.7)
I paid for the vaccination privately	56 (27.5)
*Within those who intend to get vaccinated*	*173 (100)*
**Main reason for not being vaccinated yet [only one answer]**	
Because I have to pay it out of pocket	74 (42.8)
I didn’t find the time to do it yet	28 (16.2)
My doctor didn’t recommend it to me	36 (20.8)
My doctor advised against the vaccination	6 (3.5)
Someone advised against the vaccination	3 (1.7)
I just decided during this survey that I want to get vaccinated	26 (15.0)
**How much would you be willing to pay for the HPV vaccination?** (Info: Three vaccination doses would be necessary. One vaccination dose costs approximately 160 €*)	
I would pay for the vaccination privately for the current price of about 500€	26 (15.0)
I would pay privately up to 400€	8 (4.6)
I would only get vaccinated if the vaccination is paid for by the health insurance fund	139 (80.4)
*Within those who are undecided*	*102 (100)*
**Reason(s) why you hesitate/do not want to get vaccinated [multiple answers possible]**	
I do not get vaccinated for anything	1 (1.0)
I do not consider vaccination relevant for me	19 (18.6)
HPV vaccination is too expensive for me	41 (40.2)
I am afraid of possible side effects	15 (14.7)
I do not have enough information about the HPV vaccination	43 (42.2)
Because there is no national recommendation for HPV vaccination in MSM	25 (24.5)
**How much would you be willing to pay for the HPV vaccination?** (Info: Three vaccination doses would be necessary. One vaccination dose costs approximately 160 €*)	
I would pay for the vaccination privately for the current price of about 500€	11 (10.8)
I would pay privately up to 400€	4 (3.9)
I would only get vaccinated if the vaccination is paid for by the health insurance fund	87 (85.3)
*Within those who never thought about the vaccination*	*121 (100)*
**How much would you be willing to pay for the HPV vaccination?** (Info: Three vaccination doses would be necessary. One vaccination dose costs approximately 160 €*)	
I would pay for the vaccination privately for the current price of about 500€	8 (6.6)
I would pay privately up to 400€	6 (5.0)
I would only get vaccinated if the vaccination is paid for by the health insurance fund	107 (88.4)
*Within those who refuse the HPV vaccination*	*4 (100)*
**Reason(s) why you hesitate/do not want to get vaccinated [multiple answers possible]**	
I do not consider vaccination relevant for me	3 (75.0)
I am afraid of possible side effects	1 (25.0)

* The stated approximate price of 160€ per dose refers to the time of the study conducted and may have increased meanwhile

### Source of HPV and HPV vaccination information

The most relevant source for general information about HPV was reported as internet and social media (55.0%), followed by physicians (32.6% HIV specialists, 40.9% HIV-PrEP prescribers, and 23.3% general practitioners). When asked about their preferred sources of information, participants expressed a wish for more guidance from their physicians and checkpoint centers (see supplement, Table A4). Among participants who had already heard of HPV vaccination, the primary sources of information reported were physicians (40.6% HIV-PrEP prescribers, 29.6% HIV specialists, 22.4% general practitioners), internet or social media (39.4%) and friends and relatives (21.5%) (see supplement, Table A5). Over half of these participants (53.3%) reported having received a recommendation for HPV, with the majority indicating that this recommendation was received between the ages of 18 and 26 years (56.5%). Recommendations were primarily made by physicians (55.3% HIV-PrEP prescribers, 42.2% HIV specialists and 22.7% general practitioners) and friends and relatives (13.7%). 24.5% received the vaccination due to a previous HPV infection (see supplement, Table A5).

## Discussion

In our MSM study population (*N* = 929), 21.3% had never heard of HPV before. Among those aware (78.7%), the mean HPV knowledge score showed about 66% correct answers. This indicates a general awareness of HPV among MSM; however, significant knowledge gaps persist, as evidenced by participants’ expressed need for more information regarding HPV and its associated health risks. The variability in knowledge scores, ranging from 0 to 20, and slightly lower mean scores if penalizing wrong answers, suggests that while some participants possess detailed knowledge, others may have misconceptions or gaps in understanding.

Almost two-thirds of participants stated they accept the vaccination (204 participants received at least one dose and 173 intended to get vaccinated), but only 21.0% had completed the vaccination schedule among those aware of HPV vaccination (*N* = 604; 13.7% in the total population, *N* = 929). The mean score for HPV vaccination knowledge showed a mean of 59% correct answers, indicating that while participants generally recognized the importance of vaccination, their understanding of specific aspects related to HPV vaccination could be improved. Clear, targeted information and recommendations by physicians may help address this gap. The summary scores for both HPV and HPV vaccination knowledge in our study (13.3 ± 4.3 and 3.0 ± 1.3, respectively) were comparable to those reported by Kesten et al. in 2019 who applied a similar scale to young MSM (aged 16–24 years) in England and Northern Ireland, reporting mean scores of 13.3 ± 4.7 for HPV knowledge and 3.3 ± 1.2 for HPV vaccination knowledge [16].

Missing knowledge regarding HPV and the option of prophylactic HPV vaccines was also reported in a study from 2022 evaluating knowledge and attitudes towards HPV vaccination among American college students (aged 18–25+), which found that, despite high HPV prevalence in this population, many students were misinformed about key aspects of HPV [19]. Additionally, a 2022 study revealed a significant lack of awareness of HPV and HPV vaccination in a representative sample of the general German population, with only 22.8% of male participants aware of HPV [20]. In contrast, our study found that nearly 80% of participants had at least heard of HPV. This increased awareness might be attributed to participants being generally more informed about STDs. However, the wide range of knowledge scores (0–20 for HPV knowledge; 0–5 for HPV vaccination knowledge) could indicate that high awareness does not equate to comprehensive knowledge of HPV. Further, despite a particularly high risk for HPV-associated diseases, 40.2% of all participating MSM living with HIV have not ever heard of HPV vaccination. Among those who knew about the vaccine, the vaccination knowledge score tended to be lower than in participants living without HIV, underscoring the value of tailored information within HIV care.

One of the most frequently reported reasons for vaccine hesitancy was insufficient information about HPV and vaccination, highlighting the critical role of physician recommendations in enhancing awareness. The majority of participants who were aware of HPV reported having learned about it through the internet, social media, or their physicians. Especially PrEP prescribers and HIV specialists were key sources of information, suggesting practical opportunities for targeted communication within routine care.

Two other reasons for non-vaccination were the high costs of vaccination and the absence of a national vaccination recommendation for MSM. More than 85% of hesitant participants would consider vaccination if costs were covered by their health insurance. This finding underscores the potential importance of financial accessibility in vaccination decisions, as supported by a study evaluating HPV vaccine acceptance in high-risk men in Greece, which found an association between higher income and increased willingness to vaccinate [21]. The lack of an official national recommendation for MSM might further complicate this, as it may contribute to lower perceived urgency or necessity for vaccination among this population. Other countries have already implemented national recommendations for HPV vaccination of MSM in different forms, such as Australia, Canada, Spain, UK and Italy [8–12]. They demonstrate significantly higher vaccination rates, for instance, a study from 2022 on HPV vaccination coverage in male-male partnerships in Australia found 54.2% of the study population (*N* = 1030; median age 29 years) to be vaccinated [22]. Similarly, in Italy, a study among visitors of STD clinics in 2024 reported that 41.3% of MSM (*N* = 80; mean age 36.3 years) were vaccinated against HPV [23]. These examples from other countries indicate that in the longer term, an official vaccination recommendation and the associated mandatory reimbursement by health insurers might contribute to an increase in vaccination coverage rate in this population.

Almost 70% of the vaccinated participants received the vaccination before the age of 27, which may reflect the reimbursement of the vaccination by the health insurers up to age 18 and, in some cases, voluntary reimbursement up to the age of 26 [14]. Only 30.4% of HPV vaccinations were administered in the age cohort of 27–45 years.

### Strengths and limitations

Strengths include a multicenter design that enrolled participants across Germany. The results from over 900 participants contribute to the rather scarce data landscape regarding HPV among MSM in Germany. Screening interviews conducted by physicians minimize the risk of errors in participant selection, ensuring that inclusion criteria are accurately applied. As age and HIV status can be critical to understand variations in HPV knowledge and acceptance among MSM, main results were stratified by those subgroups.

However, there are limitations. Convenience sampling through public practices specialized in men’s health/STDs including HIV likely over-represents MSM who regularly access clinical care, living with HIV, or using PrEP, and might not be representative of the broader MSM population. The urban and highly educated profile of the sample may have led to overestimation of HPV knowledge, vaccine acceptance, and uptake. Physician-mediated recruitment helped ensure eligibility and data quality but may have introduced site-specific selection effects. The 15€ time reimbursement might have influenced participation; still, recruitment was difficult and took longer than planned, suggesting only a small impact. Additionally, self-reported data can introduce recall bias, and using a no-skip format in questions on potentially sensitive topics (sexual behavior, vaccination) may have been biased towards more socially desired answers.

Further, the modules on knowledge, acceptance, and reasons were asked only among subgroups, so the corresponding estimates are conditional on awareness. To support interpretation, we consistently labeled denominators and reported uptake both for the full sample and for those aware of the vaccine. Where possible, we report „acceptance“ separately for behavior (vaccinated) and intention. The descriptive findings of the present study offer valuable insights into this important topic among MSM in Germany and may help to guide future research.

## Conclusion

About three-quarters of MSM reported prior awareness of HPV before participating in this study, correctly answering an average of 66% of statements related to HPV knowledge and 59% regarding HPV vaccination knowledge. However, the considerable variability in knowledge levels, coupled with participants expressing a desire for additional information about HPV, and the fact that 21% of participants were unaware of HPV, point to gaps in awareness and a potential need for more information regarding informed decision-making about HPV vaccination. While over 60% of participants aware of HPV vaccination expressed a willingness to get vaccinated, actual uptake among the study population was low. A combination of comprehensive information, financial reimbursement, and proactive recommendations may warrant consideration in efforts related to increased HPV vaccination uptake and protection of more MSM against certain HPV-related diseases.

## Supplementary Information

Supplementary Material 1.

## Data Availability

All data recorded by the investigator and the participants (questionnaire) were processed and stored using the electronic Case Report Form (eCRF) database (location: Alcedis GmbH, Winchesterstr. 3, 35394 Gießen, Germany). All analyses were performed by Alcedis GmbH. Wherever applicable, current guidelines of Good Pharmacoepidemiology Practice (GPP), current technical standards and guidelines were considered. The study database (eCRF database) contains all study-related data in a pseudonymized manner. The server is located in the facilities of Alcedis GmbH, Winchesterstr. 3, 35394 Gießen, Germany. Data cannot be shared publicly due to privacy restrictions.

## Abbreviations

CI: Confidence Interval
eCRF: Electronic Case Report Form
EU SPC: European Union Summary of Product Characteristics
GDPR: General Data Protection Regulation
GPP: Good Pharmacoepidemiology Practice
HIV: Human Immunodeficiency Virus
HPV: Human Papillomavirus
ICF: Informed Consent Form
MSM: Men who have sex with men
HIV-PrEP: Pre-exposure Prophylaxis for HIV
SD: Standard Deviation
STD: Sexually Transmitted Disease
STIKO: Standing Committee on Vaccination [Ständige Impfkommission]
