# Supplementary material for: HPV knowledge and acceptance of HPV vaccination among men who have sex with men (MSM) in Germany: a multicenter, observational, cross-sectional study
Source: BMC Public Health. 2025 Dec 12;26:382. doi: 10.1186/s12889-025-25746-x (PMC12853918; doi:10.1186/s12889-025-25746-x)
Supplement: Supplementary file 1 — Supplementary Material 1. [file 12889_2025_25746_MOESM1_ESM.docx]

# **Supplement**

# **Questionnaire**

**List of abbreviations**

MSM = Men who have sex with men

HPV = Human Papilloma Virus

HIV = Human Immunodeficiency Virus

PrEP = Pre-exposure Prophylaxis

AIDS = Acquired Immune Deficiency Syndrome

**Introduction to the study**

Thank you for agreeing to take part in this study on men´s knowledge and attitudes about HPV (Human Papilloma Virus). This survey will ask questions about your knowledge of HPV and what you think about HPV vaccination. It will take about 10 to 15 minutes to complete the survey.

Please answer each question below. If you are not sure of an answer, please provide your best guess. Every question has to be answered before you can go to the next question, and the survey can be completed.

**Screening questions by HCP prior to questionnaire**

Have you signed an Informed Consent Form including a Data Protection Consent Form?

1. Yes
2. No

*[If this question is answered with a), the physician will ask the following screening questions]*

Are you able to understand and answer a questionnaire in German or English?

1. Yes
2. No

Do you have sex exclusively with men?

1. Yes
2. No

Do you have sex with both men and women?

1. Yes
2. No

Do you have sex exclusively with women?

1. Yes
2. No

*[If this question was answered with a), the questionnaire ends here and the participant will not be included in the study]*

How old are you?

Age in years: ___ years

Have you lived in Germany for at least 3 years?

1. Yes
2. No

Are you insured by a German health insurance company?

1. Yes
2. No

Have you ever been tested for HIV?

1. No, I have never been tested
2. Yes, I was tested negative
3. Yes, I was tested positive

*[If this question was answered with b), the following question appears]*

When was your last test for HIV performed?

1. <3 months ago
2. 3-6 months ago
3. >6-12 months ago
4. >1 up to 3 years ago
5. >3 years ago

**If all inclusion criteria are met, the participant will be included and receive the questionnaire.**

**General questions**

***(select one answer only for each question, except if stated otherwise)***

1. Current place of residence

1. Residence with more than 500,000 inhabitants (big large city)
2. Residence with 100,000 – 500,000 inhabitants (small large city)
3. Residence with 20,000 - 100,000 inhabitants (midsized city)
4. Residence with less than 20,000 inhabitants (small city)

2. Highest educational attainment

1. No school-leaving qualification
2. Still in school/ university/ vocational training
3. Secondary school diploma (9 years of school)
4. Secondary school diploma (10 years of school)
5. General qualification for university entrance
6. Completion of vocational-in-company training (apprenticeship)/ vocational-school training (vocational college, college)
7. University/college degree

3. Which acquisition situation suits you?

1. Full-time employed
2. Part-time employed
3. Marginally employed, 520-euro job, mini-job
4. In a vocational training
5. Not employed (including pupils or students, jobseeker, early retirees)

4. How are you covered by German health insurance?

1. Statutory, compulsorily insured
2. Statutory, voluntarily insured
3. Private
4. No German health insurance

5. Which of the following categories would you place yourself in?

1. Male
2. Trans
3. Non-binary

6. What is your current relationship status?

1. Single
2. Married
3. Divorced
4. Civil Partnership
5. Separated
6. In a relationship

7. If you are ≥ 35 years: Do you take advantage of health and/or cancer check-ups (e.g. skin, colon cancer screening)? If you are <35 years: Would you take advantage of recommended check-ups?

1. Yes
2. No

8. Have you ever informed yourself about the topic of sexually transmitted diseases?

1. Yes
2. No

9. Have you ever had a sexually transmitted disease?

1. Yes
2. No
3. I do not know

10. If you have tested HIV positive, are you on therapy for this?

1. Yes
2. No

11. Have you received a pre-exposure prophylaxis (PrEP) prescription in the past?

1. Yes
2. No
3. Not yet heard of PrEP / I do not know

**Questions about sexual behavior and Human Papilloma Virus (HPV)**

12. Have you been sexually active during the last 12 months?

1. Yes
2. No

*[If question 12 is answered with b), proceed to question 17]*

13. Were men your lived sexual preference in sexual partners in the last 12 months?

1. always
2. mostly
3. sometimes
4. never

14. Number of sexual partners within the last 12 months

1. 1
2. 2 - 10
3. > 10 – 20
4. > 20

15. What sexual practices have you engaged in during the last 12 months? (select all that apply)

1. Oral
2. Anal penetrating
3. Anal receiving
4. Vaginal

16. Do you use condoms during sexual intercourse (oral and/or anal)?

1. Always
2. Mostly
3. Rarely
4. Never

17. Have you ever heard of HPV before participation in this study?

1. Yes
2. No

*[If question 17 is answered with b), questionnaire ends here]*

18. Have you ever been tested positive for HPV?

1. Yes
2. No
3. I do not know

19. Do you think that a positive HIV status increases the risk of HPV infection?

1. Yes
2. No
3. I do not know

20. Please answer the following questions to the best of your ability:

|  | True | False | I don´t know |
| --- | --- | --- | --- |
| 20.1 HPV is very rare |  |  |  |
| 20.2 HPV always has visible signs or symptoms |  |  |  |
| 20.3 HPV can be transmitted through genital skin-to-skin contact |  |  |  |
| 20.4 There are many types of HPV |  |  |  |
| 20.5 HPV can cause HIV/AIDS |  |  |  |
| 20.6 HPV can cause genital warts |  |  |  |
| 20.7 Men cannot get HPV |  |  |  |
| 20.8 Using condoms reduces the chances of HPV transmission |  |  |  |
| 20.9 HPV can be cured with antibiotics |  |  |  |
| 20.10 Having many sexual partners increases the risk of getting HPV |  |  |  |
| 20.11 HPV usually doesn't need any treatment |  |  |  |
| 20.12 Most sexually active people will get HPV at some point in  their lives |  |  |  |
| 20.13 Having sex at an early age increases the risk of getting HPV |  |  |  |
| 20.14 HPV can cause cancer in men |  |  |  |
| 20.15 HPV is a bacterial infection |  |  |  |
| 20.16 HPV can be transmitted through oral sex |  |  |  |
| 20.17 HPV can cause herpes |  |  |  |
| 20.18 HPV can be transmitted through anal sex |  |  |  |
| 20.19 HPV infections always lead to health problems |  |  |  |
| 20.20 A person with no symptoms cannot transmit the HPV infection |  |  |  |

21. Where did you get the information about HPV from? (select all that apply)

1. Pediatrician/adolescent doctor Yes/No
2. General practitioner (family doctor) Yes/No
3. Gynecologist Yes/No
4. Dermatologist Yes/No
5. Urologist Yes/No
6. HIV specialist Yes/No
7. PrEP prescriber Yes/No
8. Other doctor Yes/No
9. Public Health Service Yes/No
10. Checkpoints/counselling centers Yes/No
11. Pharmacy Yes/No
12. Friends or relatives Yes/No
13. Newspaper or magazines Yes/No
14. Radio or TV Yes/No
15. Internet or social media Yes/No
16. Other source Yes/No

22. How high would you rate your risk of getting an HPV infection?

1. High risk
2. Moderate risk
3. Low risk
4. No risk
5. I already have an infection
6. I do not know

23. Where would you go to get or how would you get (more) information about HPV? (select all that apply)

1. Pediatrician/adolescent doctor Yes/No
2. General practitioner (family doctor) Yes/No
3. Gynecologist Yes/No
4. Dermatologist Yes/No
5. Urologist Yes/No
6. HIV specialist Yes/No
7. PrEP prescriber Yes/No
8. Other doctor Yes/No
9. Public Health Service Yes/No
10. Checkpoints/counselling centers Yes/No
11. Pharmacy Yes/No
12. Friends or relatives Yes/No
13. Newspaper or magazines Yes/No
14. Radio or TV Yes/No
15. Internet or social media Yes/No
16. Other source Yes/No

**Questions about HPV vaccination and vaccination status**

24. Have you ever heard of HPV vaccination before participation in this study?

1. Yes
2. No

*[If question 24 is answered with b), questionnaire ends here]*

25. Where did you get the information about HPV vaccination from? (select all that apply)

1. Pediatrician/adolescent doctor Yes/No
2. General practitioner (family doctor) Yes/No
3. Gynecologist Yes/No
4. Dermatologist Yes/No
5. Urologist Yes/No
6. HIV specialist Yes/No
7. PrEP prescriber Yes/No
8. Other doctor Yes/No
9. Public Health Service Yes/No
10. Checkpoints/counselling centers Yes/No
11. Pharmacy Yes/No
12. Friends or relatives Yes/No
13. Newspaper or magazines Yes/No
14. Radio or TV Yes/No
15. Internet or social media Yes/No
16. Other source Yes/No

26. Has the HPV vaccination been already recommended to you?

1. Yes
2. No

*[If question 26 is answered with b), proceed to question 29]*

27. At what age were you recommended HPV vaccination?

1. 9-17 years
2. 18-26 years
3. 27-45 years

28. Where did you get the recommendation for HPV vaccination from? (*select all that apply*)

1. Pediatrician/adolescent doctor Yes/No
2. General practitioner (family doctor) Yes/No
3. Gynecologist Yes/No
4. Dermatologist Yes/No
5. Urologist Yes/No
6. HIV specialist Yes/No
7. PrEP prescriber Yes/No
8. Other doctor Yes/No
9. Public Health Service Yes/No
10. Checkpoints/counselling centers Yes/No
11. Pharmacy Yes/No
12. Friends or relatives Yes/No
13. School lessons Yes/No

29. Please answer the following questions to the best of your ability:

|  | True | False | I don´t know |
| --- | --- | --- | --- |
| 29.1 The HPV vaccines offer protection against all sexually transmitted infections |  |  |  |
| 29.2 The HPV vaccines are most effective if given to people who have never had sex |  |  |  |
| 29.3 One of the HPV vaccines offers protection against genital warts |  |  |  |
| 29.4 The HPV vaccine protects you from every type of HPV |  |  |  |
| 29.5 You can cure HPV by getting the HPV vaccine |  |  |  |

*Source: Kesten JM et al.; BMJ Open 2019*

30. Have you already been vaccinated against HPV with at least one dose?

1. Yes
2. No

*[if question 30 is answered with a), proceed to questions 31-36]*

*[if question 30 is answered with b), proceed to question 37]*

31. How many doses of HPV vaccine have you received?

1. One dose
2. Two doses
3. Three doses
4. I do not know how many doses I received but at least one

32. Are there any other HPV vaccinations planned for you?

1. Yes, still doses
2. Yes, but I don't know how many more doses
3. No
4. I don't know if there are any (more) doses planned/ needed

33. At what age were you vaccinated?

1. 9-14 years
2. 15-17 years
3. 18-26 years
4. 27-40 years
5. Over 40 years

34. Where did you receive the vaccination? (*select all that apply*)

1. Pediatrician/adolescent doctor Yes/No
2. General practitioner (family doctor) Yes/No
3. Gynecologist Yes/No
4. Dermatologist Yes/No
5. Urologist Yes/No
6. HIV specialist Yes/No
7. PrEP prescriber Yes/No
8. Other doctor Yes/No
9. Public Health Service Yes/No
10. Checkpoints/counselling centers Yes/No

35. Did you decide to get vaccinated because of a previous HPV infection/disease?

1. Yes
2. No

36. Who paid for the vaccination?

1. I was vaccinated before my 18th birthday and the vaccination was charged to my health insurance card
2. I was over 18 years old and paid for the vaccination privately but was subsequently reimbursed by my health insurance
3. I paid for the vaccination privately

37. If you have not yet been vaccinated against HPV, how do you feel about HPV vaccination?

1. I intend to get vaccinated against HPV
2. I am undecided
3. I refuse vaccination
4. I never thought about the vaccination against HPV

*[if question 37 is answered with a), proceed to question 38]*

38. What is the main reason for not being vaccinated yet? (select only one answer)

1. Because I have to pay it out of pocket
2. I didn´t find the time to do it yet
3. My doctor didn´t recommend it to me
4. My doctor advised against the vaccination
5. Someone advised against the vaccination
6. I just decided during this survey that I want to get vaccinated

*[If question 37 is answered with b) or c), proceed to question 39]*

39. Please tell us the reason(s) why you hesitate/don´t want to get vaccinated (*select all that apply*)

1. I do not get vaccinated for anything Agree/Disagree
2. I do not consider vaccination relevant for me Agree/Disagree
3. HPV vaccination is too expensive for me Agree/Disagree
4. I am afraid of possible side effects Agree/Disagree
5. I do not have enough information about the HPV vaccination Agree/Disagree
6. Because there is no national recommendation for HPV vaccination in MSM Agree/Disagree

*[If question 37 is answered with a), b) or d), proceed to questions 40 + 41]*

40. If you would be willing to get vaccinated, who do you think should do it? (*select all that apply*)

1. Pediatrician/adolescent doctor Yes/No
2. General practitioner (family doctor) Yes/No
3. Gynecologist Yes/No
4. Dermatologist Yes/No
5. Urologist Yes/No
6. HIV specialist Yes/No
7. PrEP prescriber Yes/No
8. Other doctor Yes/No
9. Public Health Service Yes/No
10. Checkpoints/counselling centers Yes/No

41. How much would you be willing to pay for the HPV vaccination? (Info: Three vaccination doses would be necessary. One vaccination dose costs approximately 160€)

1. I would pay for the vaccination privately for the current price of about 500€
2. I would pay for the vaccination privately (total costs) (please select how much you would be willing to pay):
   - up to 100 €
   - up to 200 €
   - up to 300 €
   - up to 400 €
3. I would only get vaccinated if the vaccination is paid for by the health insurance fund

# **Further Results**

**Table A1: Questions about sexual behavior and HPV**

|  | Total N (%) |
| --- | --- |
| **Analysis Population** | **929 (100)** |
| **Sexually active during the last 12 months** | |
| Yes | 913 (98.3) |
| No | 16 (1.7) |
| **Where Men your lived sexual preference in sexual partners in the last 12 months [based on sexually active men]** | |
| Always | 833 (91.2) |
| Mostly | 61 (6.7) |
| Sometimes | 19 (2.1) |
| Never | 0 (0.0) |
| **Number of sexual partners within the last 12 months [based on sexually active men]** | |
| 1 | 74 (8.1) |
| 2 - 10 | 397 (43.5) |
| > 10 - 20 | 214 (23.4) |
| > 20 | 228 (25.0) |
| **Sexual practices during the last 12 months [multiple answers possible] [based on sexually active men]** | |
| Oral | 899 (98.5) |
| Anal penetrating | 740 (81.0) |
| Anal receiving | 752 (82.4) |
| Vaginal | 57 (6.2) |
| **Use of condoms during sexual intercourse (oral and/or anal) [based on sexually active men]** | |
| Always | 56 (6.1) |
| Mostly | 296 (32.4) |
| Rarely | 400 (43.8) |
| Never | 161 (17.6) |
| **Pre-exposure prophylaxis (PrEP) prescription in the past** | |
| Yes | 717 (77.2) |
| No | 210 (22.6) |
| Not yet heard of PrEP / I do not know | 2 (0.2) |
| **Ever heard of HPV before participation in this study**^1^ | |
| Yes | 731 (78.7) |
| No | 198 (21.3) |
| **Ever tested positive for HPV [based on subjects who at least heard of HPV]** | |
| Yes | 161 (22.0) |
| No | 468 (64.0) |
| I do not know | 102 (14.0) |
| **Think that a positive HIV status increases the risk of HPV infection [based on subjects who at least heard of HPV and continued the study N=730]** | |
| Yes | 430 (58.9) |
| No | 84 (11.5) |
| I do not know | 216 (29.6) |
| **How high would you rate your risk of getting an HPV infection? [based on subjects who at least heard of HPV and continued the study N=730]** | |
| High risk | 176 (24.1) |
| Moderate risk | 251 (34.4) |
| Low risk | 163 (22.3) |
| No risk | 22 (3.0) |
| I already have an infection | 64 (8.8) |
| I do not know | 54 (7.4) |

^1^One participant discontinued study participation immediately after the “knowledge of HPV” question

**Table A2: HPV and HPV vaccination knowledge - Summary Scores – Subgroups**

[*Only for participants who had heard about HPV/HPV vaccination*]

|  | **Subgroups** | | **N** | **Mean** | **SD** | **Min** | **Q1** | **Median** | **Q3** | **Max** | **N_miss_** | **95% CI** |
| --- | --- | --- | --- | --- | --- | --- | --- | --- | --- | --- | --- | --- |
| **HPV**  **knowledge** | **Age** | **18-26 years** | 220 | 13.34 | 4.35 | 0 | 11 | 14 | 17 | 20 | 0 | 12.76; 13.91 |
|  |  | **27-45 years** | 510 | 13.26 | 4.33 | 0 | 10 | 14 | 17 | 20 | 1^1^ | 12.88; 13.64 |
|  | **HIV status** | **HIV+** | 94 | 13.00 | 4.21 | 2 | 11 | 14 | 17 | 20 | 0 | 12.14; 13.86 |
|  |  | **HIV-** | 631 | 13.35 | 4.35 | 0 | 11 | 14 | 17 | 20 | 1^1^ | 13.01; 13.69 |
|  |  | **Not tested** | 5 | 9.80 | 3.42 | 6 | 8 | 9 | 11 | 15 | 0 | 5.55; 14.05 |
|  | **Total** | **-** | 730 | 13.28 | 4.33 | 0 | 11 | 14 | 17 | 20 | 1^1^ | 12.97; 13.60 |
| **HPV vaccination knowledge** | **Age** | **18-26 years** | 191 | 3.10 | 1.37 | 0 | 2 | 3 | 4 | 5 | 0 | 2.90; 3.30 |
|  |  | **27-45 years** | 413 | 2.90 | 1.31 | 0 | 2 | 3 | 4 | 5 | 0 | 2.78; 3.03 |
|  | **HIV status** | **HIV+** | 73 | 2.60 | 1.34 | 0 | 2 | 3 | 4 | 5 | 0 | 2.29; 2.92 |
|  |  | **HIV-** | 528 | 3.02 | 1.32 | 0 | 2 | 3 | 4 | 5 | 0 | 2.91; 3.14 |
|  |  | **Not tested** | 3 | 1.33 | 1.15 | 0 | 0 | 2 | 2 | 2 | 0 | -1.54; 4.20 |
|  | **Total** | **-** | 604 | 2.97 | 1.33 | 0 | 2 | 3 | 4 | 5 | 0 | 2.86; 3.07 |

Abbreviations: CI, Confidence Interval; HIV, Human Immunodeficiency Virus; HPV, Human Papillomavirus; Max, Maximum; Min, Minimum; N_miss_, Number of missings; SD, Standard Deviation; Q1, first quartile; Q3, third quartile.

^1^One participant discontinued study participation immediately after the “knowledge of HPV” question and before answering any of the HPV knowledge statements. Therefore, no summary score could be calculated for this participant.

**Table A3: HPV and HPV vaccination knowledge - Summary Scores – Subgroups – Sensitivity analysis**

[*Alternative calculation method*]

|  | **Subgroups** | | **N** | **Mean** | **SD** | **Min** | **Q1** | **Median** | **Q3** | **Max** | **N_miss_** | **95% CI** |
| --- | --- | --- | --- | --- | --- | --- | --- | --- | --- | --- | --- | --- |
| **HPV knowledge** | **Age** | **18-26 years** | 220 | 10.94 | 5.21 | -3 | 8 | 11 | 15 | 20 | 0 | 10.25; 11.63 |
|  |  | **27-45 years** | 510 | 10.85 | 5.13 | -3 | 7 | 11 | 15 | 20 | 1 | 10.40; 11.30 |
|  | **HIV status** | **HIV+** | 94 | 10.22 | 5.08 | -3 | 6 | 11 | 14 | 20 | 0 | 9.18; 11.26 |
|  |  | **HIV-** | 631 | 11.01 | 5.16 | -3 | 7 | 11 | 15 | 20 | 1 | 10.60; 11.41 |
|  |  | **not tested** | 5 | 7 | 3.54 | 2 | 5 | 8 | 9 | 11 | 0 | 2.61; 11.39 |
|  | **Total** | **-** | 730 | 10.88 | 5.15 | -3 | 7 | 11 | 15 | 20 | 1 | 10.50; 11.25 |
| **HPV vaccina-tion knowledge** | **Age** | **18-26 years** | 191 | 2.5 | 1.85 | -2 | 1 | 3 | 4 | 5 | 0 | 2.23; 2.76 |
|  |  | **27-45 years** | 413 | 2.28 | 1.73 | -3 | 1 | 2 | 4 | 5 | 0 | 2.11; 2.45 |
|  | **HIV status** | **HIV+** | 73 | 1.84 | 1.86 | -2 | 1 | 2 | 3 | 5 | 0 | 1.40; 2.27 |
|  |  | **HIV-** | 528 | 2.43 | 1.74 | -3 | 1 | 3 | 4 | 5 | 0 | 2.28; 2.58 |
|  |  | **not tested** | 3 | 1 | 1.73 | -1 | -1 | 2 | 2 | 2 | 0 | -3.30; 5.30 |
|  | **Total** | **-** | 604 | 2.35 | 1.77 | -3 | 1 | 2 | 4 | 5 | 0 | 2.21; 2.49 |

Abbreviations: CI, Confidence Interval; HIV, Human Immunodeficiency Virus; HPV, Human Papillomavirus; Max, Maximum; Min, Minimum; Nmiss, Number of missings; SD, Standard Deviation; STD, Sexually Transmitted Disease; Q1, first quartile; Q3, third quartile.

**Table A4: Questions about HPV information**

[*Only for participants who had heard about HPV*]

| **Questions about HPV information** | **Total N (%)** |
| --- | --- |
| Number of participants who had already heard of HPV | 731 (100.00) |
| **Question 21: Information about HPV from [multiple answers possible]** | |
| Pediatrician/adolescent doctor | 24 (3.28) |
| General practitioner (family doctor) | 170 (23.26) |
| Gynecologist | 12 (1.64) |
| Dermatologist | 62 (8.48) |
| Urologist | 57 (7.80) |
| HIV specialist | 238 (32.56) |
| PrEP prescriber | 299 (40.90) |
| Other doctor | 64 (8.76) |
| Public Health Service | 44 (6.02) |
| Checkpoints/counselling centers | 83 (11.35) |
| Pharmacy | 17 (2.33) |
| Friends or relatives | 194 (26.54) |
| Newspaper or magazines | 50 (6.84) |
| Radio or TV | 50 (6.84) |
| Internet or social media | 402 (54.99) |
| Other source | 131 (17.92) |
| **Question 22: How high would you rate your risk of getting an HPV infection?** | |
| High risk | 176 (24.08) |
| Moderate risk | 251 (34.34) |
| Low risk | 163 (22.30) |
| No risk | 22 (3.01) |
| I already have an infection | 64 (8.76) |
| I do not know | 54 (7.39) |
| Missing | 1 (0.14) |
| **Question 23: Where would you go to get or how would you get (more) information about HPV? [multiple answers possible]** | |
| Pediatrician/adolescent doctor | 20 (2.74) |
| General practitioner (family doctor) | 354 (48.43) |
| Gynecologist | 39 (5.34) |
| Dermatologist | 112 (15.32) |
| Urologist | 189 (25.85) |
| HIV specialist | 447 (61.15) |
| PrEP prescriber | 507 (69.36) |
| Other doctor | 88 (12.04) |
| Public Health Service | 153 (20.93) |
| Checkpoints/counselling centers | 259 (35.43) |
| Pharmacy | 58 (7.93) |
| Friends or relatives | 68 (9.30) |
| Newspaper or magazines | 21 (2.87) |
| Radio or TV | 18 (2.46) |
| Internet or social media | 306 (41.86) |
| Other source | 47 (6.43) |

Abbreviations: HIV, Human Immunodeficiency Virus; HPV, Human Papillomavirus; PrEP, Pre-exposure Prophylaxis.

**Table A5: Questions about HPV vaccination**

[*Only for participants who had heard about HPV vaccination*]

| **Questions about HPV vaccination** | **Total N (%)** |
| --- | --- |
| Number of participants who did get information about HPV vaccination | 604 (100.00) |
| **Question 25: Information about HPV vaccination from**  **[multiple answers possible]** | |
| Pediatrician/adolescent doctor | 27 (4.47) |
| General practitioner (family doctor) | 135 (22.35) |
| Gynecologist | 7 (1.16) |
| Dermatologist | 24 (3.97) |
| Urologist | 19 (3.15) |
| HIV specialist | 179 (29.64) |
| PrEP prescriber | 245 (40.56) |
| Other doctor | 45 (7.45) |
| Public Health Service | 33 (5.46) |
| Checkpoints/counselling centers | 43 (7.12) |
| Pharmacy | 13 (2.15) |
| Friends or relatives | 130 (21. 52) |
| Newspaper or magazines | 33 (5.46) |
| Radio or TV | 58 (9.60) |
| Internet or social media | 238 (39.40) |
| Other source | 74 (12.25) |
| **Question 26: HPV vaccination already recommended** | |
| Yes | 322 (53.31) |
| No | 282 (46.69) |
| **Question 27: Age when recommended HPV vaccination**  **[based on participants who had already been recommended]** | |
| 9-17 years | 29 (9.01) |
| 18-26 years | 182 (56.52) |
| 27-45 years | 111 (34.47) |
| **Question 28: Recommendation for HPV vaccination from [multiple answers possible] [based on participants who had already been recommended]** | |
| Pediatrician/adolescent doctor | 13 (4.04) |
| General practitioner (family doctor) | 73 (22.67) |
| Gynecologist | 2 (0.62) |
| Dermatologist | 14 (4.35) |
| Urologist | 10 (3.11) |
| HIV specialist | 136 (42.24) |
| PrEP prescriber | 178 (55.28) |
| Other doctor | 31 (9.63) |
| Public Health Service | 10 (3.11) |
| Checkpoints/counselling centers | 30 (9.32) |
| Pharmacy | 2 (0.62) |
| Friends or relatives | 44 (13.66) |
| School lessons | 8 (2.48) |

Abbreviations: HIV, Human Immunodeficiency Virus; HPV, Human Papillomavirus; PrEP, Pre-exposure Prophylaxis.

# **Ethics Committees**

An ethics vote for this study was obtained in accordance with local legal requirements for non-interventional studies in Germany prior to the start of data collection. The study protocol was reviewed and initially approved on 18 SEP 2023 by the ethics committee Ärztekammer Berlin, Berlin, Germany (central ethics committee), and was then submitted to all local ethics committees. Any amendments to the study protocol were submitted to the central as well as local ethics committees of each participating site.

All Ethics Committees involved are listed in **Table** B1, including respective submission and approval dates of the different protocol versions.

**Table B1: Ethics Committees**

| **Site number** | **Site name** | **City** | **Name of Ethics Committee** | **Reference number** | **Submission Date** | **Approval Date** |
| --- | --- | --- | --- | --- | --- | --- |
| 01 | Novopraxis Berlin GbR | Berlin | Ärztekammer Berlin, Berlin, Germany | Eth-33/23 | 02.06./17.08./ 02.10.2023/ 03.04.2024 (Amendment01) | 18.09.2023/ 09.10.2023/  22.05.2024 |
| 02 | prinzmed | München | -^1^ | -^1^ | -^1^ | -^1^ |
| 03 | St. Elisabeth-Hospital | Bochum | EC Westfalen Lippe | 2023-575-b-S | 18.10.2023/ 27.05.2024 (Amendment01) | 19.10.2023/ 10.06.2024 |
| 04 | MVZ Clotten Zweigpraxis | Freiburg | Landesärztekammer Baden-Württemberg | B-F-2023-103 | 18.10.2023/ 27.05.2024 (Amendment01) | 14.11.2023/ 25.06.2024 |
| 05 | Arztpraxis Dr. Rößler | Zwickau | Landesärztekammer Sachsen | EK-BR-122/23-1 | 23.10.2023/ 27.05.2024 (Amendment01) | 07.11.2023/ 28.05.2024 |
| 06 | Praxenzentrum Blondelstrasse Dr. Knechten | Aachen | EC Nordrhein | 2023244 | 11.12.2023/ 27.05.2024 (Amendment01) | 14.12.2023/ 29.05.2024 |
| 07 | MVZ ZIMIH Wünsche. Berlin | Berlin | Ärztekammer Berlin, Berlin, Germany | Eth-33/23 | 11.12.2023/ 03.04.2024 (Amendment01) | 13.12.2023/ 22.05.2024 |
| 08 | Dr. Scholten & Schneeweiß GbR | Köln | EC Nordrhein | 2023244 | 26.10.2023/ 27.05.2024 (Amendment01) | 14.12.2023/ 29.05.2024 |
| 09 | Universitäts-klinikum Hamburg-Eppendorf | Hamburg | Landesärztekammer Hamburg | 2023-200881-BO-bet | 15.12.2023/ 08.01.2024/ 27.05.2024 (Amendment01) | 21.12.2023/ 15.01.2024/ 28.05.2024 |
| 10 | Universitäts-klinikum Essen | Essen | EC Essen | 23-11537-BO | 18.10.2023/ 27.05.2024 (Amendment01) | 16.01.2024/ 31.05.2024 |

Data Source: Data extracted from the study database.

^1^The ethics committee in Munich followed the decision of the central ethics committee Ärztekammer Berlin.
